# County Health Leadership Practices and Readiness for Noncommunicable Disease Services in Kenya

**DOI:** 10.5334/aogh.2673

**Published:** 2022-07-22

**Authors:** Paul Wekesa, Kevin Owuor, Cheryl Beers Cullen

**Affiliations:** 1 Centre for Health Solutions – Kenya (CHS), 4th Floor, CVS Plaza, Kasuku road off Lenana road, P.O. Box 23248-00100 GPO, Nairobi, Kenya; 2 Centre for Health Solutions – Kenya (CHS), KE; 3 Walden University, College of Health Sciences, US

**Keywords:** Noncommunicable diseases, service readiness, leadership practices, county, Kenya

## Abstract

**Background:**

Premature mortality from noncommunicable diseases (NCDs) is a contemporary development challenge. Low-income and lower-middle-income countries are disproportionately affected, with the poorest in society considered the most vulnerable. A paucity of literature exists on how leadership practices at the implementation level relate to ensuring readiness for NCD services.

**Objective:**

This study investigated any relationship between leadership practices and readiness for NCD services.

**Methods:**

This correlational study investigated any relationship between leadership practices at the county level and readiness for NCD services in Kenya using secondary data from a 2013 Service Availability and Readiness Assessment survey. Correlation and multiple linear regression tests were used to determine the strength and direction of any relationship between leadership practices (annual work planning, therapeutic committees, and supportive supervision), and NCD readiness (county readiness score).

**Findings:**

The findings indicated a statistically significant relationship between therapeutic committee (*p* = .002) and supportive supervision practices (*p* = .023) and NCD readiness. Leadership practices also had a statistically significant predictive relationship with NCD readiness (*p* = .009).

**Conclusion:**

Health leaders should ensure that leadership practices that have a predictive relationship with NCD readiness, such as therapeutic committee activities and supportive supervision visits, are implemented appropriately. Further, county health leaders should pay particular attention to the implementation of these leadership practices at nonpublic and Tiers 2, 3, and 4 health facilities that had lower NCD readiness scores.

## Introduction

Worldwide, noncommunicable diseases (NCDs) resulted in 70% of all deaths in 2019. Three quarters of these deaths occurred in low- and middle-income countries, including 82% of the 16 million premature mortalities before the age of 70 in 2019 [1]. Given the growing and disproportionate global burden, NCDs are argued to be an important global social justice issue [2].

In 2012, the World Health Assembly targeted a 25% reduction in preventable deaths from NCDs by 2025. The focus of this effort has been on cardiovascular diseases, cancers, diabetes, and chronic respiratory diseases, which cause 87% of all NCD deaths [3]. In 2015, reduction of premature NCD mortality was also prioritized as a sustainable development goal [4]. Recent arguments have advanced the need to reconceptualize NCDs as socially transmitted conditions to recognize societal factors as determinants of NCDs beyond individual-level factors [2,5,6]. These developments emphasize health services for NCDs as a contemporary leadership issue.

Health facilities in sub-Saharan Africa were historically designed to manage acute conditions from infectious, maternal or neonatal diseases [7,8]. Yet the burden of disease attributable to chronic NCDs is growing and projected to exceed that from acute infectious diseases requiring chronic care [7,9]. This epidemiological transition, is amplified by demographic shifts from population growth and aging with an increasing number of people requiring chronic disease management for NCDs seeking health services [7,9]. This occurs in the backdrop of a health system whose capacity is generally weak, fragmented and varying in quality with limited projected increments in health spending in the next 25 years [7,10,11].

In Kenya, there is evidence of a growing NCD epidemic [12]. The World Health Organization (WHO) reported an increase in NCD-related mortality in Kenya, from 99 630 deaths to 120 000 between 2012 and 2019 [13,14]. An estimated 21% of these deaths affected adults below 70 years of age and were considered premature and largely preventable [13,15]. Years of healthy life lost due to disability or mortality caused by NCDs, expressed as disability-adjusted life years (DALYs), were 6.54 million in 2015 [14,16].

About half of the hospital admissions in Kenya and 39% of all deaths were NCD-related [17,18]. By 2025, it is projected that NCD-related admissions will exceed those from communicable diseases, denoting an epidemiological transition [9]. Most of these admissions occur at tertiary health facilities [18]. Because out-of-pocket payments for health care are most common in Kenya, most households are vulnerable to being exposed to poverty as a result of NCDs [17,19].

Limitations exist in NCD care in Kenya. Screening for NCDs is suboptimal [18]. More than half of the adult population had never received a blood pressure measurement to evaluate cardiovascular risk [23]. Three out of every 4 cases of diabetes remain undiagnosed and contributed to late presentation with end-organ complications of diabetes [24,25]. When diagnosed, treatment is limited by inadequate access to oncologists, radiotherapy services, treatment services for infectious causes of cancer, and cost [26,27,28,29]. Because of these barriers, 80% of cancer patients are diagnosed with advanced disease, contributing to poor disease outcomes and mortality [26]. For conditions such as hypertension, successful treatment is limited by low adherence because of unavailability of medication, low perception of risk and cost, yet investing in prevention and treatment could avert between 249 and 391 DALYs and reduce the cost of patient care [26,30,31,32,33]. While chronic obstructive pulmonary disease (COPD) was the commonest cause of mortality from respiratory causes in 2015, there is limited investment in the management of COPD, and it remains under-researched [34]. Similar to other East African countries, gaps in human resources for health because of shortages as well as knowledge deficits limit access and scale-up of NCD care [11,12,32].

County health leaders in Kenya participate in routine leadership activities such as work planning, therapeutic committee activities and supportive supervision. Every year, county departments of health are required to prepare annual work plans that identify priority activities and allocate resources for the next fiscal year [20]. County health leaders also supervise multidisciplinary therapeutic committees that oversight the use of medicines, health products and technologies [21]. At various intervals, county health leaders also conduct supportive supervision visits to health facilities focused on improving health service coverage and quality [22]. There is however, a paucity of literature on whether any relationship exists between such leadership practices and readiness for NCD services at implementation level, which is the focus of this study.

## Methods

### Data source

This study used data from the Service Availability and Readiness Assessment (SARA) conducted in Kenya. The SARA is a comprehensive health facility assessment that utilizes standard indicators and measures to evaluate service availability and readiness of the health sector [35,36]. The SARA can be used to evaluate valid, reliable, and standardized assessment of general (infrastructure, personnel, and utilization) and specific (health interventions) service availability as well as overall capacity to provide basic and specific health services (readiness) [35]. This national census was conducted by the Kenyan ministry of health, the WHO and the U.S. Agency for International Development (USAID) in 2013.

### Study setting

All departments of health drawn from the 47 subnational county governments in Kenya participated in the survey. County governments through their departments of health are responsible for the delivery of health services in Kenya [37]. Assessed health facilities were selected from an existing master facility list that contains facility identification information, including the name, address, and geolocation codes of all public, private for-profit, private non-profit, and faith-based health facilities within each county. The master facility list also includes information such as staffing, number of hospital beds, and services provided at each facility [35,38]. The SARA data from the 47 counties contained results from 7 396 out of 8 401 health facilities with majority located in rural areas. Table 1 presents county health facilities by location, tier, and ownership.

**Table 1:** County health facilities by location, type, and ownership.


COUNTY	N	LOCATION		TYPE OF FACILITY				MANAGING AUTHORITY (OWNERSHIP)				
		
		URBAN N (%)	RURAL N (%)	TIER 1 N (%)	TIER 2 N (%)	TIER 3 N (%)	TIER 4 N (%)	GOVERNMENT/PUBLIC N (%)	MISSION/FAITH-BASED N (%)	NGO/PRIVATE NOT-FOR-PROFIT N (%)	OTHER N (%)	PRIVATE FOR-PROFIT N (%)

Baringo	188	161 (87.5)	23 (12.5)	7 (3.7)	22 (11.7)	145 (77.1)	14 (7.4)	151 (80.3)	13 (6.9)	6 (3.2)	5 (2.7)	13 (6.9)

Bomet	113	111 (99.1)	1 (0.9)	5 (4.4)	14 (12.4)	91 (80.5)	3 (2.7)	97 (85.8)	5 (4.4)	7 (6.2)	1 (0.9)	3 (2.7)

Bungoma	149	120 (80.5)	29 (19.5)	16 (10.7)	20 (13.4)	85 (57)	28 (18.8)	94 (63.1)	12 (8.1)	8 (5.4)	8 (5.4)	27 (18.1)

Busia	83	72 (86.7)	11 (13.3)	10 (12)	15 (18.1)	49 (59)	9 (10.8)	60 (72.3)	12 (14.5)	3 (3.6)	1 (1.2)	7 (8.4)

Elgeyo-Marakwet	123	118 (95.9)	5 (4.1)	9 (7.3)	20 (16.3)	90 (73.2)	4 (3.3)	104 (84.6)	13 (10.6)	0 (0)	0 (0)	6 (4.9)

Embu	150	127 (86.4)	20 (13.6)	10 (6.7)	12 (8)	77 (51.3)	51 (34)	76 (50.7)	22 (14.7)	10 (6.7)	0 (0)	42 (28)

Garissa	133	68 (51.1)	65 (48.9)	15 (11.3)	23 (17.3)	41 (30.8)	54 (40.6)	71 (53.4)	0 (0)	6 (4.5)	1 (0.8)	55 (41.4)

Homa Bay	182	166 (91.7)	15 (8.3)	18 (9.9)	41 (22.5)	99 (54.4)	24 (13.2)	121 (66.5)	24 (13.2)	19 (10.4)	4 (2.2)	14 (7.7)

Isiolo	42	34 (81)	8 (19)	4 (9.5)	6 (14.3)	27 (64.3)	5 (11.9)	26 (61.9)	8 (19)	2 (4.8)	1 (2.4)	5 (11.9)

Kajiado	226	116 (51.3)	110 (48.7)	46 (20.4)	31 (13.7)	79 (35)	70 (31)	78 (34.5)	22 (9.7)	19 (8.4)	4 (1.8)	103 (45.6)

Kakamega	232	197 (85.3)	34 (14.7)	30 (12.9)	37 (15.9)	98 (42.2)	67 (28.9)	120 (51.7)	28 (12.1)	9 (3.9)	5 (2.2)	70 (30.2)

Kericho	174	153 (88.4)	20 (11.6)	13 (7.5)	12 (6.9)	133 (76.4)	16 (9.2)	132 (75.9)	10 (5.7)	14 (8)	3 (1.7)	15 (8.6)

Kiambu	352	219 (62.8)	130 (37.2)	83 (23.6)	40 (11.4)	143 (40.6)	86 (24.4)	89 (25.3)	56 (15.9)	34 (9.7)	3 (0.9)	170 (48.3)

Kilifi	231	131 (57)	99 (43)	15 (6.5)	15 (6.5)	86 (37.2)	115 (49.8)	90 (39)	8 (3.5)	15 (6.5)	13 (5.6)	105 (45.5)

Kirinyaga	142	102 (72.3)	39 (27.7)	31 (21.8)	11 (7.7)	61 (43)	39 (27.5)	50 (35.2)	28 (19.7)	9 (6.3)	0 (0)	55 (38.7)

Kisii	141	114 (80.9)	27 (19.1)	24 (17)	31 (22)	69 (48.9)	17 (12.1)	101 (71.6)	16 (11.3)	5 (3.5)	2 (1.4)	17 (12.1)

Kisumu	155	103 (66.5)	52 (33.5)	29 (18.7)	35 (22.6)	70 (45.2)	21 (13.5)	89 (57.4)	16 (10.3)	21 (13.5)	8 (5.2)	21 (13.5)

Kitui	322	273 (85)	48 (15)	20 (6.2)	36 (11.2)	214 (66.5)	52 (16.1)	240 (74.5)	20 (6.2)	5 (1.6)	3 (0.9)	54 (16.8)

Kwale	95	86 (90.5)	9 (9.5)	7 (7.4)	9 (9.5)	56 (58.9)	23 (24.2)	62 (65.3)	4 (4.2)	6 (6.3)	1 (1.1)	22 (23.2)

Laikipia	93	67 (73.6)	24 (26.4)	6 (6.5)	10 (10.8)	59 (63.4)	18 (19.4)	56 (60.2)	8 (8.6)	11 (11.8)	4 (4.3)	14 (15.1)

Lamu	43	33 (76.7)	10 (23.3)	5 (11.6)	4 (9.3)	23 (53.5)	11 (25.6)	25 (58.1)	4 (9.3)	1 (2.3)	0 (0)	13 (30.2)

Machakos	299	232 (80.8)	55 (19.2)	13 (4.3)	31 (10.4)	136 (45.5)	119 (39.8)	141 (47.2)	28 (9.4)	14 (4.7)	5 (1.7)	111 (37.1)

Makueni	193	159 (82.8)	33 (17.2)	18 (9.3)	24 (12.4)	111 (57.5)	40 (20.7)	118 (61.1)	24 (12.4)	7 (3.6)	1 (0.5)	43 (22.3)

Mandera	69	45 (65.2)	24 (34.8)	10 (14.5)	19 (27.5)	27 (39.1)	13 (18.8)	49 (71)	1 (1.4)	0 (0)	1 (1.4)	18 (26.1)

Marsabit	93	74 (81.3)	17 (18.7)	10 (10.8)	15 (16.1)	51 (54.8)	17 (18.3)	57 (61.3)	14 (15.1)	0 (0)	1 (1.1)	21 (22.6)

Meru	405	259 (67.3)	126 (32.7)	36 (8.9)	30 (7.4)	126 (31.1)	213 (52.6)	119 (29.4)	51 (12.6)	6 (1.5)	4 (1)	225 (55.6)

Migori	174	140 (80.9)	33 (19.1)	23 (13.2)	27 (15.5)	97 (55.7)	27 (15.5)	112 (64.4)	19 (10.9)	17 (9.8)	1 (0.6)	25 (14.4)

Mombasa	373	4 (1.1)	359 (98.9)	30 (8)	20 (5.4)	54 (14.5)	269 (72.1)	51 (13.7)	11 (2.9)	29 (7.8)	23 (6.2)	259 (69.4)

Muranga	184	165 (90.2)	18 (9.8)	14 (7.6)	17 (9.2)	117 (63.6)	36 (19.6)	106 (57.6)	24 (13)	9 (4.9)	2 (1.1)	43 (23.4)

Nairobi	876	4 (0.5)	860 (99.5)	134 (15.3)	95 (10.8)	116 (13.2)	531 (60.6)	98 (11.2)	53 (6.1)	109 (12.4)	39 (4.5)	577 (65.9)

Nakuru	328	220 (67.1)	108 (32.9)	35 (10.7)	45 (13.7)	144 (43.9)	104 (31.7)	133 (40.5)	40 (12.2)	42 (12.8)	10 (3)	103 (31.4)

Nandi	190	177 (93.2)	13 (6.8)	6 (3.2)	20 (10.5)	143 (75.3)	21 (11.1)	128 (67.4)	17 (8.9)	18 (9.5)	4 (2.1)	23 (12.1)

Narok	135	117 (87.3)	17 (12.7)	9 (6.7)	25 (18.5)	91 (67.4)	10 (7.4)	89 (65.9)	24 (17.8)	10 (7.4)	3 (2.2)	9 (6.7)

Nyamira	114	98 (86)	16 (14)	15 (13.2)	39 (34.2)	42 (36.8)	18 (15.8)	69 (60.5)	15 (13.2)	10 (8.8)	0 (0)	20 (17.5)

Nyandarua	116	97 (85.1)	17 (14.9)	14 (12.1)	20 (17.2)	50 (43.1)	32 (27.6)	59 (50.9)	13 (11.2)	4 (3.4)	0 (0)	40 (34.5)

Nyeri	252	164 (65.9)	85 (34.1)	46 (18.3)	20 (7.9)	91 (36.1)	95 (37.7)	101 (40.1)	22 (8.7)	4 (1.6)	2 (0.8)	123 (48.8)

Samburu	77	59 (77.6)	17 (22.4)	5 (6.5)	6 (7.8)	52 (67.5)	14 (18.2)	45 (58.4)	11 (14.3)	5 (6.5)	1 (1.3)	15 (19.5)

Siaya	158	142 (89.9)	16 (10.1)	13 (8.2)	37 (23.4)	91 (57.6)	17 (10.8)	118 (74.7)	16 (10.1)	7 (4.4)	4 (2.5)	13 (8.2)

Taita Taveta	81	68 (84)	13 (16)	7 (8.6)	16 (19.8)	40 (49.4)	18 (22.2)	55 (67.9)	4 (4.9)	2 (2.5)	2 (2.5)	18 (22.2)

Tana River	49	44 (89.8)	5 (10.2)	3 (6.1)	4 (8.2)	40 (81.6)	2 (4.1)	40 (81.6)	4 (8.2)	1 (2)	0 (0)	4 (8.2)

Tharaka Nithi	117	95 (81.2)	22 (18.8)	7 (6)	11 (9.4)	70 (59.8)	29 (24.8)	57 (48.7)	23 (19.7)	3 (2.6)	4 (3.4)	30 (25.6)

Trans-Nzoia	141	88 (62.4)	53 (37.6)	12 (8.5)	12 (8.5)	45 (31.9)	72 (51.1)	52 (36.9)	14 (9.9)	9 (6.4)	1 (0.7)	65 (46.1)

Turkana	142	112 (80)	28 (20)	8 (5.6)	18 (12.7)	92 (64.8)	24 (16.9)	94 (66.2)	26 (18.3)	1 (0.7)	0 (0)	21 (14.8)

Uasin Gishu	170	108 (67.1)	53 (32.9)	16 (9.4)	24 (14.1)	96 (56.5)	34 (20)	99 (58.2)	23 (13.5)	9 (5.3)	3 (1.8)	36 (21.2)

Vihiga	87	78 (89.7)	9 (10.3)	11 (12.6)	18 (20.7)	26 (29.9)	32 (36.8)	44 (50.6)	10 (11.5)	6 (6.9)	2 (2.3)	25 (28.7)

Wajir	113	67 (59.3)	46 (40.7)	16 (14.2)	27 (23.9)	39 (34.5)	31 (27.4)	76 (67.3)	2 (1.8)	3 (2.7)	0 (0)	32 (28.3)

West Pokot	96	84 (87.5)	12 (12.5)	5 (5.2)	8 (8.3)	67 (69.8)	16 (16.7)	56 (58.3)	20 (20.8)	2 (2.1)	2 (2.1)	16 (16.7)


### Independent variables

This included variables measuring availability of annual work plans, quarterly therapeutic committee meetings, and supervision visits from health leaders. The first independent variable measured whether management had the annual work plans for the period 2012 to 2013. The second measured whether quarterly medicine and therapeutics committee meetings were held by health leaders in the previous 12 months, and the third measured whether health leaders had conducted at least 4 supervision visits to the facility in the previous twelve months. These variables collected at the health-facility level were aggregated to a county-level score presented as a standardized percentage score on a 0–100 scale.

### Dependent variable

The dependent variable was the county NCD Readiness Index score presented as a percentage score on a standardized 0–100 scale for each county. The county NCD readiness index was an aggregate score derived from the availability of staff (to diagnose and manage specific NCDs), guidelines (national guidelines for the management of specific NCDs), training (of staff on the diagnosis and management of specific NCDs in the last two years), and equipment (availability and functionality of equipment for management of specific NCDs on the day of the visit).

## Analysis

SPSS version 24 was used for data analysis. The SPSS software was used to analyze the independent and dependent variables to investigate any relationship between the variables. Initial analysis included frequencies and descriptive statistics. Correlation analysis was used to analyze relationships between variables, and multiple linear regression analysis was used to test the predictive model. The level of statistical significance accepted for all tests was *p* ≤ .05.

## Limitations

Limitations for this study align with the limitations of secondary analysis. The available data used to investigate the research questions and hypotheses were not specifically collected for this purpose. Content validity issues relating to the extent to which survey instruments represented study concepts could apply.

Additionally, other variables that might have been useful for this study may have been omitted. Similar to other studies using SARA data, only three NCDs were included in data collection, omitting others such as cancers, mental, and neurological conditions [11]. The selection of variables was based on the available variables collected during primary data collection. The selection of predictor variables for this study was also limited to the three considered most relevant. With this limitation, other potentially confounding variables were not included in this study. Moreover, the reliability of the findings and conclusions of this study was dependent on the rigor of the methods used in collecting the primary data.

## Study results

Table 2 presents descriptive statistics including mean and standard deviation for the independent and dependent variables. Annual work planning had the highest mean, and quarterly medicine and therapeutics committee meetings had the lowest. Based on the means, it appeared that health leaders performed medicine and therapeutic committee activities least.

**Table 2:** Descriptive statistics for the independent and dependent variables.


	*N*	MEAN	STD. DEVIATION

Independent variables			

Medicine and therapeutics committees	47	16.74	9.124

Annual work planning	47	63.89	15.646

Supervisory visits	47	62.62	15.159

Dependent variable			

NCD readiness	47	44.06	16.263

NCD readiness by tier of county health facilities			

Readiness by Tier 1	47	57.23	21.697

Readiness by Tier 2	47	47.70	17.865

Readiness by Tier 3	47	36.11	15.287

Readiness by Tier 4	47	23.70	11.786

NCD readiness by ownership of county health facilities			

Ownership—Public	47	41.02	16.426

Ownership—Private not-for-profit	47	35.19	13.925

Ownership—Private for-profit	47	24.23	12.532

Ownership—Other	47	17.51	25.079


The mean county NCD readiness score was 44.06. County health facilities categorized as Tier 1 (hospitals) had the highest mean NCD readiness, whereas Tier 4 health facilities (other stand-alone facilities) had the least. Based on ownership, publicly owned county health facilities had the highest mean NCD readiness scores, whereas those categorized as *other* had the lowest scores.

Correlation analysis was used to investigate any conjectured relationship between each leadership practice variable—namely, annual work planning, therapeutic committees, and supportive supervision—and whether any relationship existed with NCD readiness. Pearson correlation coefficient tests were performed in SPSS for each variable pair. As shown in Table 3, quarterly medicine and therapeutic committees and supportive supervision correlated significantly with the dependent variable, NCD readiness. Annual work planning as a leadership practice did not correlate significantly with NCD readiness.

**Table 3:** Correlations of Leadership Practices and NCD Readiness.


CORRELATIONS					

		NCD READINESS	MEDICINE AND THERAPEUTIC	ANNUAL WORK PLAN	SUPPORTIVE SUPERVISION

NCD readiness	Pearson correlation	1	.438**	.286	.330*

	Sig. (2 tailed)		.002	.051	.023

	*N*	47	47	47	47

Medicine and therapeutic	Pearson correlation	.438**	1	.629**	.625**

	Sig. (2 tailed)	.002		.000	.000

	*N*	47	47	47	47

Annual work plan	Pearson correlation	.286	.629**	1	.833**

	Sig. (2 tailed)	.051	.000		.000

	*N*	47	47	47	47

Supportive supervision	Pearson correlation	.330*	.651**	.833**	1

	Sig. (2 tailed)	.023	.000	.000	

	*N*	47	47	47	47


* Correlation is significant at the 0.05 level (2-tailed). ** Correlation is significant at the 0.01 level (2-tailed).

A multiple linear regression analysis was conducted to investigate the predictive role of leadership practices namely annual work planning, therapeutic committees, and supportive supervision on the dependent variable, county NCD readiness. The set of predictor variables was related to county NCD readiness at a statistically significant level, F(3,3.519) = 43, *p* < .05, *R*^2^ = .197, Adj. *R*^2^ = .141).

Both quarterly medicine and therapeutic committee activities and supportive supervision visits were positively related at a statistically significant level to county NCD readiness. Quarterly medicine and therapeutics committee activities (β = .438) was however a better predictor than supportive supervision (β = .330) as shown in Table 4.

**Table 4:** Linear Model of Predictors of NCD Readiness.


MODEL	*SE B*	β	95% CONFIDENCE INTERVAL FOR *B*	

			LOWER BOUND	UPPER BOUND

Medicine and therapeutic	.438	.438**	.300	1.262

Annual work plan	.286	.286	–.002	.596

Supportive Supervision	.330	.330*	.050	.658

*R* ^2^	.197			

*F*	3.519*			


* *p* < .05. ** *p* < .01.

## Discussion

The aim was to understand health leadership practices at the county level and how these relate to readiness for NCD services. The results indicated a statistically significant positive correlation for leadership practices relating to medicine and therapeutic committee activities, supportive supervision, and county NCD readiness.

Literature exists on the relationship between leadership and health outcomes. For NCD services, whereas literature focuses on leadership actions at the political and global levels, there is a paucity of research on leadership actions and practices at the implementation level [39]. This study focused primarily on leadership practices at the implementation level.

This study demonstrated a relationship between leadership and NCD services that aligns with existing literature that posits the importance of prioritizing leadership interventions for NCD response [40,41,42].

Wong argued that supportive leadership approaches and practices were related to positive patient outcomes [43]. Although this study did not focus on direct patient outcomes, findings supported a positive relationship between supportive leadership practices and readiness for health services. Specifically, Vasan et al. (2017) argued that there were positive effects of leadership practices such as supportive supervision on health services including immunization, management of childhood diarrhea, and malaria services [44]. This study adds to the literature on the positive relationship between supportive supervision practices and NCD service readiness.

### Policy Implications

The positive social change implications of this study include providing evidence that could be used by county departments of health to evaluate their leadership practices and how they relate to readiness for NCD services. Through such county self-assessments, it would be possible to ensure prioritization of practices most aligned to achieving readiness for NCD services. The findings of this study could also be used to develop capacity-building programs targeting health leaders at the county level.

The results of this study indicate that therapeutic committee and supportive supervision activities had a positive relationship with readiness for NCD services. However, what was implemented was not aligned, in that therapeutic committee activities were least practiced by county departments of health. At the individual level, health leaders could use the findings of this study to reflect on their own leadership practices and how they relate to readiness for NCD services in their counties.

Prioritizing leadership practices and actions for NCD readiness could have benefits for society. A better understanding of the leadership role could support the prioritization of actions aimed at proximal access to NCD services. Where this occurs, improved readiness for NCD services would ensure access to required prevention and treatment services.

## Conclusions

County departments of health should review existing leadership practices to prioritize those that correlate with NCD readiness. Health leaders should also ensure that leadership practices that have a predictive relationship with NCD readiness, such as therapeutic committee activities and supportive supervision visits, are implemented appropriately. Further, county health leaders should pay particular attention to the implementation of these leadership practices at nonpublic and Tiers 2, 3, and 4 health facilities that had lower NCD readiness scores. This is the first analysis investigating leadership practices at implementation level and NCD readiness in Kenya, and despite the limitations, the findings could be used to enhance practice and to further investigate ways to enhance county readiness for NCDs and other health services.

## Additional File

The additional file for this article can be found as follows:

10.5334/aogh.2673.s1 NCD readiness dataset. This dataset contains data analysis used in this study.

## Author Information

We, the above listed authors, certify that we have participated sufficiently in the conception and design of this manuscript and the analysis of the data, as well as writing of the manuscript, and to take public responsibility for it. The manuscript represents valid and original work and reflects no plagiarism issues. We have reviewed the final version of the manuscript and approve it for publication. Neither has the manuscript or one with substantially similar content under my/our authorship been published or is being considered for publication elsewhere. We certify that all the data collected during the study is presented in this manuscript and no data from the study has or will be published separately.
